# UHPLC-Q-Orbitrap HRMS and network analysis to explore the mechanisms of QiShenYiQi dripping pill for treating myocardial infarction

**DOI:** 10.3389/fphar.2024.1443560

**Published:** 2024-11-01

**Authors:** Zhichao Liu, Huanjie Fu, Yongkang Gan, Yujia Ye, Binghui Huang, Mingxiu Jiang, Jinhong Chen, Xiaofeng Li

**Affiliations:** ^1^ School of Rehabilitation Medicine, Shandong Second Medical University, Weifang, Shandong, China; ^2^ Department of Cardiovascular, Second Teaching Hospital of Tianjin University of Traditional Chinese Medicine, Tianjin, China; ^3^ Department of Vascular Surgery, Tianjin Academy of Traditional Chinese Medicine Affiliated Hospital, Tianjin, China

**Keywords:** myocardial infarction, QiShenYiQi dripping pills, NO-cGMP-PKG, systems pharmacology, network analysis

## Abstract

This study focused on examining the protection of QiShenYiQi dripping pills (QSYQ) against myocardial infarction (MI) and investigating its potential mechanisms. Ultra high performance liquid chromatography-q exactive-orbitrap high resolution mass spectrometry (UHPLC-Q-Orbitrap HRMS) was employed to analyze potential active compounds of QSYQ. The targets of these compounds were predicted using an integrated *in silico* method and cross-referenced with relevant databases to identify associated pathways. Experimental validation was then conducted to confirm the accuracy of the systems pharmacology findings. In the end, network analysis combined with UHPLC screened 13 potential active compounds and obtained 99 targets for the intersection of potential active compounds and diseases. The enrichment analysis results indicated that the cyclic guanosine monophosphate-protein kinase G (cGMP-PKG) signaling pathway might be the mechanism of action of QSYQ in the treatment of MI. Experimental verification demonstrated that QSYQ could alleviate oxidative stress, promote vasodilation, and activate proteins related to the mitochondrial ATP-sensitive potassium channel (K_ATP_) and nitric oxide (NO)-cGMP-PKG signaling pathway. This study provides insights into both the pathogenic mechanisms underlying MI and the molecular mechanisms through which QSYQ may confer protection. Given the role of PKG in regulating myocardial stiffness, it emerges as a promising therapeutic target for myocardial remodeling. We propose that the NO-cGMP-PKG and mitochondrial K_ATP_ pathways may serve as candidate therapeutic targets for the development of new interventions for MI.

## 1 Introduction

From the perspective of pathology, myocardial infarction is myocardial cell death resulting from extended ischemia (Thygesen et al., 2018). It shows the typical manifestations of chest pain, discomfort, and acute shortness of breath (Reed et al., 2017). Even though post-myocardial infarction (MI) cardiovascular mortality is greatly reduced during 2000–2017, there has been an obvious increase in the risk of non-cardiovascular morbidity. Clearly, non-cardiovascular causes occupy a critical part in post-MI mortality. Based on the above results, more attention should be paid to non-cardiovascular outcomes in clinical practice and guidelines, which must be considered in designing future clinical studies (Christensen et al., 2023). In recent decades, the development of pharmacology and the application of percutaneous coronary intervention (PCI) make significant contributions to improving patient prognosis (Hoole and Bambrough, 2020; Damluji et al., 2021). However, immediate reperfusion can exacerbate myocardial injury, which is called myocardial ischemia-reperfusion injury (Algoet et al., 2023). Reperfusion can deteriorate myocardial ischemia/reperfusion injury, causing cardiac dysfunction including myocyte death, reperfusion arrhythmia, myocardial stunning, endothelial/microvascular dysfunction, like inflammatory response, no-reflow phenomenon, or severe myocardial tissue injuries compared with those resulting from primary ischemic insult (Xiang et al., 2021). The application of PCI does not represent the final stage in treating MI. Despite the associated risks, PCI remains the cornerstone of the treatment approach. This is of particular importance for those with great infarcts or those with no early revascularization, as they continue to face an increased risk of mortality (Dhruva et al., 2020).

Therefore, there is significant interest in the development of effective strategies to improve the efficacy and prognosis of MI treatment. Complementary and alternative therapeutic approaches, particularly those rooted in traditional Chinese medicine (TCM), have gained attention as a potential source of innovative treatments. TCM plays a vital role in Chinese healthcare and is increasingly recognized in modern medicine for its potential in drug discovery. Despite the growing prominence of TCM in treating major clinical diseases, its broader clinical adoption is hindered by challenges including ambiguous drug composition or treatment mechanisms that may result in restricted clinical improvement (Qi et al., 2023). QiShenYiQi dripping pills (QSYQ) is the standardized TCM medication that approved by the China Food and Drug Administration (Approval Number of CFDA: Z20030139) for treating cardiovascular disease in 2003 (Zhang et al., 2012). QSYQ has been used in managing heart failure (Chang et al., 2019), MI (Wang et al., 2017), and ischemia-reperfusion injury (Han et al., 2019). It is consisted of *Astragalus membranaceus* Fisch. ex Bunge [Fabaceae; Astragali radix], *Salvia miltiorrhiza* Bunge [Lamiaceae; Salviae miltiorrhizae radix et rhizoma], *Panax notoginseng* (Burkill) F. H. Chen [Araliaceae; Notoginseng radix et rhizome], and *Dalbergia odorifera* T. Chen [Fabaceae; Dalbergiae odoriferae lignum]at a ratio of 10:5:1:0.067 (Huang et al., 2021a). The use of QSYQ can reduce primary and secondary endpoints of MI patients, and improve the quality of life without inducing adverse events (Bai et al., 2023).

Some studies have explored the mechanism of QSYQ in relieving MI by using network pharmacology and transcriptome analysis methods. According to gene expression data analysis and literature mining, one study has identified 12 major compounds in QSYQ that exhibit the potential to regulate various pathways by modulating groups of genes, thereby making the anti-MI effects. These compounds are implicated in essential biological processes including anti-inflammation, anti-apoptosis, antioxidation, anti-coagulation, acceleration of angiogenesis, and facilitation of energy use (Li et al., 2014). The fundamental research has demonstrated that QSYQ exhibits diverse therapeutic impacts on MI. It effectively reduces the progression of ventricular remodeling, alleviates inflammation attributed to the arachidonic acid lipoxygenase pathway and nitric oxide (NO) production, and improves abnormalities in blood lipid levels (Wang et al., 2017). Although QSYQ has been shown to be capable of inhibiting microvascular endothelial inflammation and activating NO-cyclic guanosine monophosphate-protein kinase G (cGMP-PKG) pathway in heart failure mice (Huang et al., 2021b), the identification of potential active ingredients and their potential to improve MI are not fully understood.

Research have demonstrated that the bioactive components of botanical drugs are linked to the substances and compounds that enter the bloodstream following oral administration and subsequent metabolism. Serum pharmacochemistry has emerged as a practical approach for investigating the active ingredients responsible for the therapeutic effects of TCM. This methodology has gained significant recognition and widespread application (Tang et al., 2022). Network analysis, which employs network-based methodologies, explores the complex interactions between drugs, diseases, and targets while considering the various components, targets, and pathways involved. This approach facilitates a comprehensive understanding of the effectiveness and underlying mechanisms of different TCM constituents from a holistic perspective. Its use has been extensively applied in contemporary TCM research (Li and Zhang, 2013). Therefore, to further elucidate the cardioprotective mechanisms of QSYQ, the comprehensive pharmacology method was used to explore the pharmacological mechanisms related to QSYQ in MI treatment. Briefly, the ultra high performance liquid chromatography-q exactive-orbitrap high resolution mass spectrometry (UHPLC-Q-Orbitrap HRMS) approach was constructed to identify potential effective components of QSYQ. On this basis, a compound-target network was built to predict candidate targets by topology analysis. Subsequently, the targets acquired were aligned in databases to elucidate the associated pathways. Follow-up experiments were also performed to validate the accuracy of predictions based on bioinformatics analysis and molecular biology methods. These comprehensive results shed valuable lights on the clinical indications of QSYQ and its constituent drug components, explaining their potential therapeutic benefits in the context of MI.

## 2 Materials and methods

### 2.1 Main reagents

QSYQ was kindly provided by Tasly Pharmaceutical Group Co. Ltd. (Batch number: 230432, Tianjin, China). Isosorbide 5-mononitrate (ISMN) was obtained from AstraZeneca Pharmaceutical Group Co. Ltd. (Batch number: 2206081, Wuxi, China). Formic acid, methanol, and acetonitrile and water were LC-MS grade and were purchased from Fisher Scientific (Waltham, MA, United States). Test kits for creatine kinase (CK) and lactate dehydrogenase (LDH) were purchased from Dirui Bioengineering (Changchun, China). ELISA kits were acquired as follows: malondialdehyde (MDA) activity assay kit and Glutathione (GSH) kit from Jiancheng (Jiangsu, China), superoxide dismutase (SOD) activity assay kit from Beyotime (Shanghai, China), nitrite kit from Aidisheng (Jiangsu, China), and cGMP kit from Jianglai (Shanghai, China). Antibodies against enzyme nitric oxide synthase (eNOS), Kir6.1, and protein kinase C epsilon (PKC-epsilon) were purchased from Abcam (Cambridge, England). Kir6.2 and PKG were purchased from Thermo Fisher Scientific (Waltham, MA, United States). The secondary antibody was purchased from Affinity (Jiangsu, China).

### 2.2 Chemical components and serum pharmacochemistry study of QSYQ

#### 2.2.1 Animal preparation

Sprague Dawley (SD) rats (male, 180–220 g, 6 weeks old) were obtained from SPF (Beijing) Biotechnology Co., Ltd. (Beijing, China). This study was carried out following Guide for the Care and Use of Laboratory Animals (NIH, 8th Edition, 2011). The experimental protocol was approved by the Experimental Animal Ethics Committee of Tianjin Jinke Bona Biotechnology Company Limited (Approval No. GENINK20230015).

After 1 week of adaptive feeding, 12 rats were weighed. Nine of them were randomly assigned to receive QSYQ solution (540 mg/kg dissolved in 0.9% normal saline, corresponding to four times the clinically equivalent dose). Three rats were sampled at 0.5, 1, and 2 h, respectively. After anesthesia, blood was drawn into serum tubes, and then, immediately centrifuged at 3,500 rpm and 4°C to obtain serum for UHPLC-Q-Orbitrap HRMS. Blank serum samples were prepared using the same method.

#### 2.2.2 Solution preparation

One gram of QSYQ was combined with 40 mL 80% methanol and subjected to ultrasonic extraction for 30 min. The suspension was then transferred and centrifuged at 4°C for 10 min at 12,000 rpm. The supernatant was collected for subsequent analysis. For blank serum and drug serum samples, they were mixed with methanol and vortexed for 10 min. The mixture was then centrifuged at 4°C for 10 min at 12,000 rpm. The supernatant was concentrated using vacuum centrifugation for 4 h. Following this, a 50% methanol-water solution was added, and the mixture was vortexed for 1 min. The supernatant was then transferred for analysis. The corresponding standards were dissolved in methanol. All the above sample solutions are stored in a refrigerator at 4°C and protected from light.

#### 2.2.3 Chromatographic and mass spectrometric methods

QSYQ solution, serum sample solution, and standard solution were analyzed under identical conditions using UHPLC-Q-Orbitrap HRMS. The chromatographic and mass spectrometric methods were as follows:

Separation was performed using a Vanquish Flex UHPLC system (Thermo Fisher Scientific, Waltham, MA, United States) equipped with an ACQUITY UPLC HSS T3 column (2.1 mm × 100 mm, 1.8 μm particle size; Waters, MA, United States). The injection volume was 10 μL. The mobile phase was consisted of acetonitrile with 0.1% formic acid (phase A) and water with 0.1% formic acid (phase B) at a flow rate of 0.3 mL/min, with the column maintained at 40°C. The gradient elution program was: 0–1.0 min (2%–2% A); 1.0–41.0 min (2%–100% A); 41.0–50.0 min (100%–100% A); 50.0–52 min (100%–2% A).

MS data was acquired by a hybrid quadrupole orbitrap mass spectrometer (Q-Exactive, Thermo Fisher Scientific, Waltham, MA, United States) equipped with a HESI-II spray probe. The parameters were set as follows: positive ion source voltage at 3.7 kV, negative ion source voltage at 3.5 kV, heated capillary temperature at 320°C, sheath gas pressure at 30 psi, auxiliary gas pressure at 10 psi, and desolvation temperature at 300°C. Nitrogen was used as both the sheath and auxiliary gas, as well as the collision gas at a pressure of 1.5 mTorr. Data were acquired in “Full scan/dd-MS^2^” mode with full scan parameters set to a resolution of 7,000, an auto gain control target of 1 × 10^6^, and a maximum isolation time of 50 ms. The dd-MS^2^ data were collected with a resolution of 17,500, an auto gain control target of 1 × 10^5^, a maximum isolation time of 50 ms, a loop count of the top 10 peaks, an isolation window of m/z 2, collision energies of 10 V, 30 V, 60 V, and an intensity threshold of 1 × 10^5^.

#### 2.2.4 Data processing and compound identification

MS data was processed using Progenesis QI 3.0 (Waters, MA, United States) through steps including raw data import, peak extraction, and adduct deconvolution. Identification was finalized by considering retention time (Rt) error of the reference substance, mass error of the parent ion, match degree of fragment ions, and isotope distribution. These parameters were cross-referenced with the reference substance database (TCM Pro 2.0, Beijing Hexin Technology Co., Ltd.) and a theoretical database constructed from literature and public sources. Based on the analysis of QSYQ components, a database comprising the mass-to-charge ratios (*m/z*) of QSYQ components and their Phase I and Phase II metabolites was constructed. This database was then applied to analyze the absorbed components in the blood. Initially, the *m/z* values were retrieved using Progenesis QI, ensuring that the Rts of these *m/z* values were close to those of the corresponding components in QSYQ solution, and that the signals of these *m/z* values in the medicated blood were at least ten times higher than those in the blank blood. This strategy enabled the comprehensive analysis of both QSYQ components and their absorbed components.

### 2.3 Network analysis

#### 2.3.1 Screening of potential active components and targets of QSYQ

Botanical drug emphasizes the synergy of multiple compounds and targets in treating complex disorders (Yang et al., 2022). In this study, the compounds identified in rat serum could be the potential active components of QSYQ. A comprehensive *in silico* approach was employed to identify potential targets for these potential active components. The TCMSP (https://tcmsp-e.com/) (Ru et al., 2014), SwissTarget Prediction (http://www.swisstargetprediction.ch/) (Gfeller et al., 2014), and DrugBank (https://www.drugbank.ca/) (Wyse et al., 2018) databases were employed to predict target proteins of QSYQ.

#### 2.3.2 Screening of MI genes

GeneCards database (https://www.genecards.org/) (Stelzer et al., 2016) and Online Mendelian Inheritance in Man (OMIM, https://omim.org/) (Amberger et al., 2015) were applied to obtain known anti-MI therapeutic targets.

#### 2.3.3 Network establishment and topological analysis

Network establishment enables us to uncover the regulatory principles of small molecules efficiently in a high-throughput fashion. We mapped QSYQ-associated targets with MI-associated targets, aiming to determine their shared candidate targets using the Venn diagram. Afterwards, a compound-target network was constructed with Cytoscape 3.8.0 for understanding the complicated compound-target interactions at a systemic level. Subsequently, topology analysis was completed using a network analyzer (Assenov et al., 2008) to extract the core hub network including main components and key targets.

#### 2.3.4 Functional enrichment

Metascape (http://metascape.org/) (Zhou et al., 2019), an online platform, includes the integrative gene list annotation and analysis for the experimental research, and can also be used for Kyoto Encyclopedia of Genes and Genomes (KEGG) and Gene Ontology (GO) enrichment analyses. Key targets were uploaded into Metascape for generating GO-biological process, cellular component, molecular function, and KEGG pathway analyses, with the parameter being set as “H species.” Then, we selected the most significant GO and KEGG results based on the pharmaceutical and physiological importance. Typical terms enriched were visualized via bioinformatics (http://www.bioinformatics.com.cn).

#### 2.3.5 Modular analysis via the molecular complex detection (MCODE) algorithm

MCODE analyzes the network in line with vertex weighting through local neighborhood density and outward traversal from the locally-dense seed protein, aiming to isolate dense regions based on the provided parameters. MCODE (Version 1.4.2) (Bader and Hogue, 2003) was utilized for identifying the highly associated network components among common potential targets. Thereafter, functional enrichment of MCODE components was performed using Metascape, while function descriptions of the corresponding modules were arranged according to the p-value.

### 2.4 *In vivo* experimental study of QSYQ in improving MI

#### 2.4.1 Animal model and drug administration

SPF SD male rats (180–220 g) were obtained from SPF (Beijing) Biotechnology Co., Ltd. (Beijing, China). All rats were maintained in the pathogen-free environment, under 22°C ± 2°C, 50%–70% of relative humidity, and the 12-h/12-h light/dark cycle. Rats had free access to water and food.

Left anterior descending (LAD) coronary artery of rats was ligated to induce the MI model. Briefly, the heart was exposed through making a chest incision, followed by ligation of left coronary artery 1.5–2 mm beneath the left atrial appendage. The thorax was then closed layer by layer. The rats in the sham group received the similar procedure but had no actual ligation in LAD. Rats were kept warm using the heated blanket postoperatively. In the end, we successfully obtained 35 MI model rats and 7 sham-operation rats. One day after the surgery, rats with LAD ligation were randomly divided into 5 groups (7 per group) including model group, ISMN group, low, middle, and high-dose QSYQ groups.

ISMN has been frequently used in the America as the long-acting NO donor; besides, it is also commonly applied in the treatment of coronary artery disease (Rassaf and Kelm, 2013). Actually, early administration of nitrates during acute MI may curtail infarct expansion and hinder left ventricular dilation (Pipilis et al., 1993). The combination of delayed reperfusion and ISMN in acute anterior MI can expedite the recovery of ventricular function and mitigate cardiac remodeling (Jugdutt et al., 1995). As a result, ISMN was selected to be the positive control agent. ISMN and QSYQ at equal dosages were determined based on body surface area (Xu, 2002). After calculation, rats of low, middle, and high-dose QSYQ groups received concentrated QSYQ at dosages of 135, 270, and 540 mg/kg, while those of positive control group received concentrated ISMN at 5.4 mg/kg, and those of model and sham-operated groups received water at an equal amount. After 28 days of continuous administration, echocardiography was conducted in each rat after overnight fasting. Thereafter, the rats were sacrificed, and blood and the heart were collected, with tissues being preserved in liquid nitrogen prior to the subsequent analysis.

#### 2.4.2 Echocardiography

The Vevo2100 ultrahigh-resolution ultrasound system (VisualSonics, Canada) was used in standard transthoracic echocardiogram analysis before and following a 28-day drug intervention. Animals were placed after isoflurane anesthesia (2.5% induction and 2.0% maintenance with oxygen) in the process of transthoracic echocardiography. The parasternal long axis view was captured through B-mode echocardiography. The left ventricular parameters, including fractional shortening (FS) and ejection fraction (EF), were assessed.

#### 2.4.3 Creatine kinase and lactate dehydrogenase measurements

Blood biochemical testing was performed within 12 h following the successful preparation of the rat MI model. CK and LDH contents in serum were determined with commercial kits using the automatic biochemistry analyzer (Mindray, Shenzhen, China). Data were represented by international units (IU)/L.

#### 2.4.4 Colorimetry and ELISA

Myocardial tissue at its apical part was collected and homogenized into the 5% suspension. To measure the levels of oxidative stress, MDA activity assay kit and SOD activity assay kit were used following manufacturers’ protocols. GSH and nitrite expression in myocardial tissue homogenates were detected differently using kits, following the manufacturer’s protocols. Nitrate within the sample could be reduced into nitrite with nitrate reductase. Meanwhile, nitrite level was determined with sodium nitrite being the reference. Optical density (OD) values were detected with the spectrophotometer. We defined enzyme activity unit as decomposition of 1 μmol H_2_O_2_ to one enzyme activity unit/g of sample within the 37°C water bath. Protein extraction kits were employed to extract myocardial tissue proteins beneath the ligature. The mixed sample was subjected to homogenization and 10-min centrifugation at 3,000 rpm and 4°C, and the resulting supernatant was collected as the total protein. Later, ELISA was utilized to assess the plasma cGMP content with the microplate reader according to the specific protocols.

#### 2.4.5 Western blot assay

The protein lysis buffer was added to lyse myocardial tissue for 30 min on ice under ultrasonic treatment. The BCA protein assay kit was used to measure protein content in tissue lysate supernatants. Then, protein separation was completed with 8% sodium dodecyl sulphate polyacrylamide gel electrophoresis, followed by transfer onto polyvinylidene fluoride membranes. After blocking with 5% defatted milk for a 2-h period, membranes were probed using primary antibodies overnight under 4°C, including eNOS (1:1,000), Kir6.1 (1:1,000), Kir6.2 (1:1,000), PKC-epsilon (1:1,000), PKG (1:1,000), and anti-GAPDH (1:2,000) antibodies, followed by another 1-h incubation using horseradish peroxidase-labeled secondary antibody (1:4,000) under room temperature. Bands were visualized using Bands were visualized using enhanced chemiluminescence luminescent reagent, while an automated gel imaging system was performed with the exposure imaging approach. Protein bands were determined by Image Lab software. Luminescent reagent, while an automated gel imaging system was performed with the exposure imaging approach. Protein bands were determined by Image Lab software.

### 2.5 Statistical analysis

Data were represented by mean ± standard error of the mean (SEM). Student’s t-test (two-sided) was used to compare two groups, whereas one-way analysis of variance (ANOVA) plus Newman–Keuls *post hoc* test was applied to compare multiple groups. GraphPad Prism 8.0 (GraphPad Software, Inc., La Jolla, CA, United States) was employed for statistical analysis, with *P* < 0.05 representing statistical significance.

## 3 Result

### 3.1 Chemical components analysis of QSYQ

To elucidate the major chemical components in QSYQ, UHPLC-Q-Orbitrap HRMS was developed and applied. The base peak ions chromatograms for positive and negative modes are shown in Supplementary Figure S1. A total of 111 components in QSYQ were identified, mainly flavonoids, triterpenoid saponins, nucleosides, phenolic acids, amino acids, and other compounds. The identification was based on comparisons of Rt error, mass error of the parent ion, match degree of fragment ions, and isotope distribution with those of reference standard compounds and reported data. The mass error for all identified compounds was within ±5 ppm. A summary of the identified constituents and their corresponding data is provided in Supplementary Table S1.

### 3.2 Identification of components absorbed into the blood from QSYQ

**FIGURE 1:**
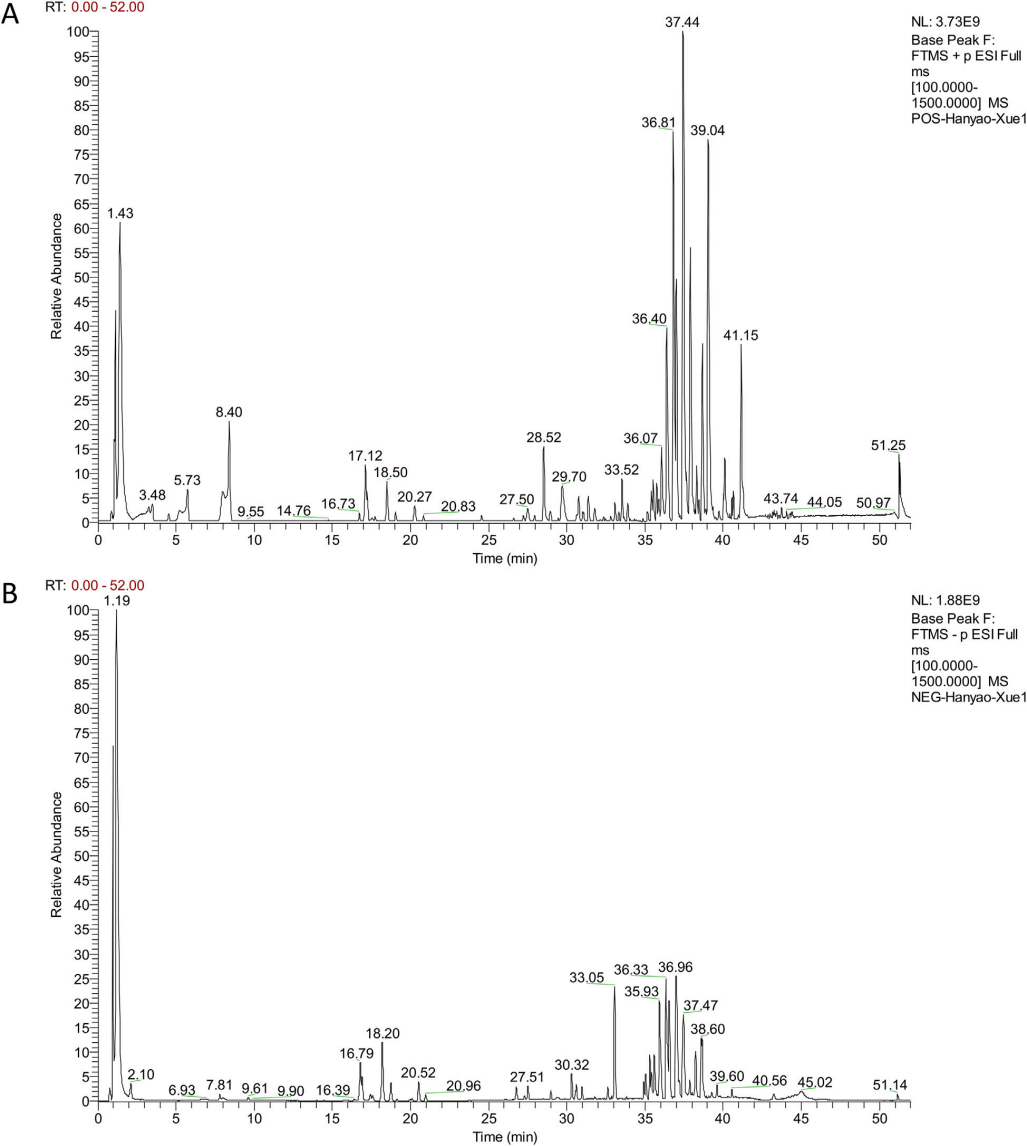
The base peak ions of QSYQ-containing serum detected in positive **(A)** and negative **(B)** modes.

**FIGURE 2:**
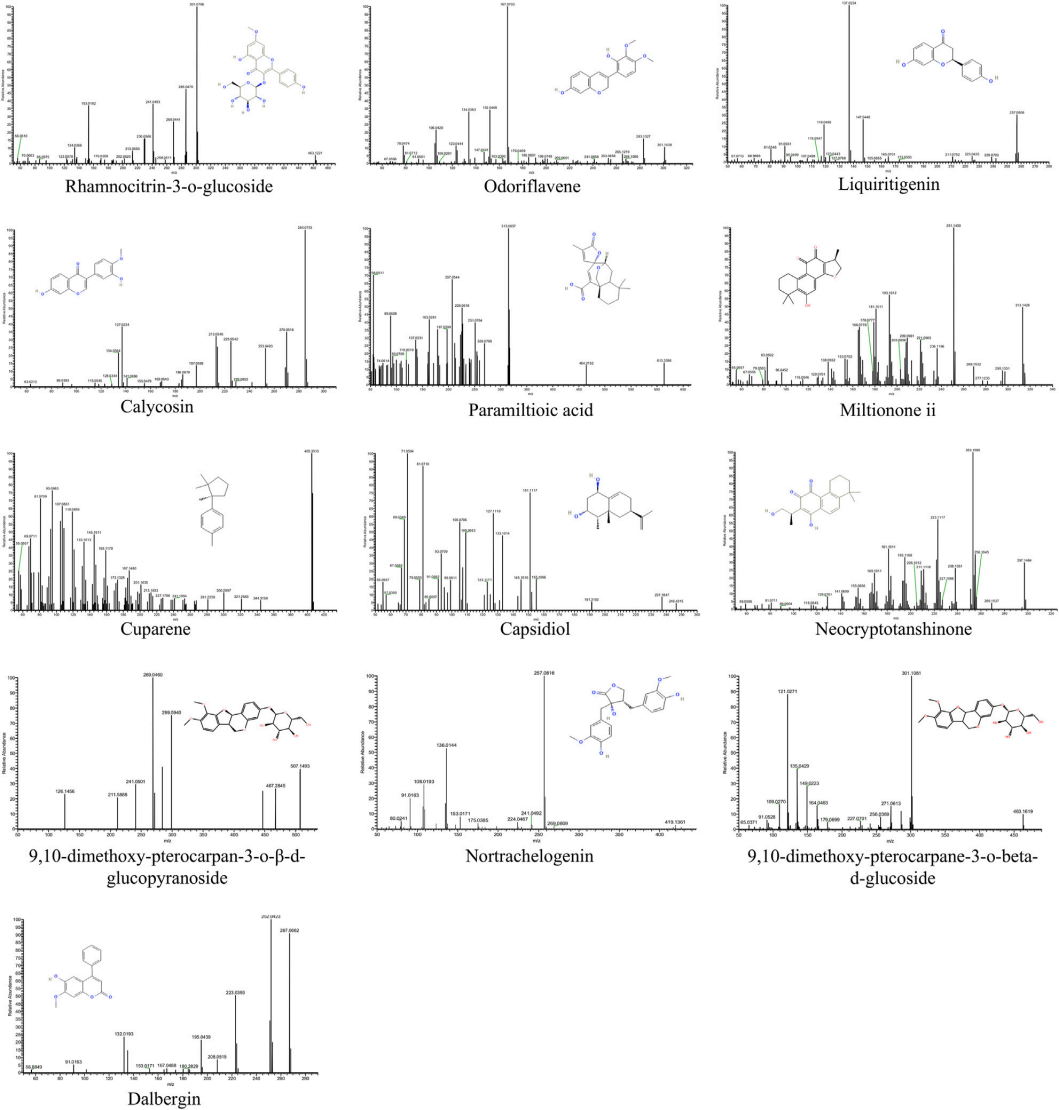
The structures and their corresponding MS/MS spectra of 13 potential active compounds.

Since QSYQ exerts its therapeutic effects after being digested and entering circulation (Tang et al., 2022), we further studied the serum chemistry of rats in QSYQ-containing and blank groups (Supplementary Figure S2). According to the method described in Section 2.2.4, a total of 17 differentially expressed components, including 4 prototypes and 13 metabolites, were identified in the QSYQ-containing serum. Its base peak ions chromatograms and identification results are shown in Figure 1 and Table 1. The prototype and metabolite components detected in serum were arisen from 13 chemical components of QSYQ measured in Section 3.1. Figure 2 illustrates the structures along with their corresponding MS/MS spectra of these 13 components, which are regarded as potential active compounds of QSYQ in this investigation.

**TABLE 1:** Main components in QSYQ-containing serum samples.

No.	Class	Parent compound	Transformation	Molecular formula	Rt/min	*m/z*	Error (ppm)	Adducts	Herb
1	Prototype	Odoriflavene		C_17_H_16_O_5_	20.59	301.1063	2.35	[M + H]^+^	4
2	Prototype	Miltionone II		C_19_H_20_O_4_	25.75	313.1427	2.39	[M + H]^+^	2
3	Prototype	Neocryptotanshinone		C_19_H_22_O_4_	30.39	297.1478	2.47	[M + H-H_2_O]^+^	2
4	Prototype	Paramiltioic acid		C_19_H_24_O_5_	32.58	313.1446	0.33	[M-H-H_2_O]^-^	2
5	Metabolite	Calycosin	+C_6_H_8_O_6_	C_16_H_12_O_5_	18.98	459.0939	1.33	[M-H]^-^	1
6	Metabolite	9,10-dimethoxy-pterocarpane-3-o-beta-d-glucoside	+C_6_H_8_O_6_	C_23_H_28_O_10_	19.09	639.1939	1.28	[M-H]^-^	1
7	Metabolite	Dalbergin	+C_6_H_8_O_6_	C_16_H_12_O_4_	19.75	443.0987	0.73	[M-H]^-^	4
8	Metabolite	9,10-dimethoxy-pterocarpan-3-o-β-d-glucopyranoside	+O-H_2_	C_23_H_26_O_10_	20.16	475.1248	0.38	[M-H]^-^	1
9	Metabolite	Rhamnocitrin-3-o-glucoside	+O-H_2_	C_22_H_22_O_11_	20.81	475.0884	0.51	[M-H]^-^	1
10	Metabolite	Cuparene	-H_4_	C_15_H_22_	21.54	199.1480	0.53	[M + H]^+^	3
11	Metabolite	Capsidiol	-H_2_+O_2_	C_15_H_24_O_2_	22.59	271.1898	2.32	[M + H]^+^	1
12	Metabolite	Miltionone II	-CO	C_19_H_20_O_4_	24.06	285.1478	2.6	[M + H]^+^	2
13	Metabolite	Nortrachelogenin	-COO	C_20_H_22_O_7_	25.31	329.1397	0.69	[M-H]^-^	2
14	Metabolite	Liquiritigenin	-COO	C_15_H_12_O_4_	28.23	213.0905	2.27	[M + H]^+^	4
15	Metabolite	Neocryptotanshinone	-CO	C_19_H_22_O_4_	28.69	287.1632	3.42	[M + H]^+^	2
16	Metabolite	Miltionone II	-H_4_	C_19_H_20_O_4_	29.52	309.1113	2.75	[M + H]^+^	2
17	Metabolite	Miltionone II	+H_2_	C_19_H_20_O_4_	33.11	315.1579	3.91	[M + H]^+^	2

1: *Astragalus membranaceus* Fisch. ex Bunge [Fabaceae; Astragali radix]; 2: *Salvia miltiorrhiza* Bunge [Lamiaceae; Salviae miltiorrhizae radix et rhizoma]; 3: *Panax notoginseng* (Burkill) F. H. Chen [Araliaceae; Notoginseng radix et rhizome]; 4: *Dalbergia odorifera* T. Chen [Fabaceae; Dalbergiae odoriferae lignum].

### 3.3 Network analysis of the potential active compounds in QSYQ

**FIGURE 3:**
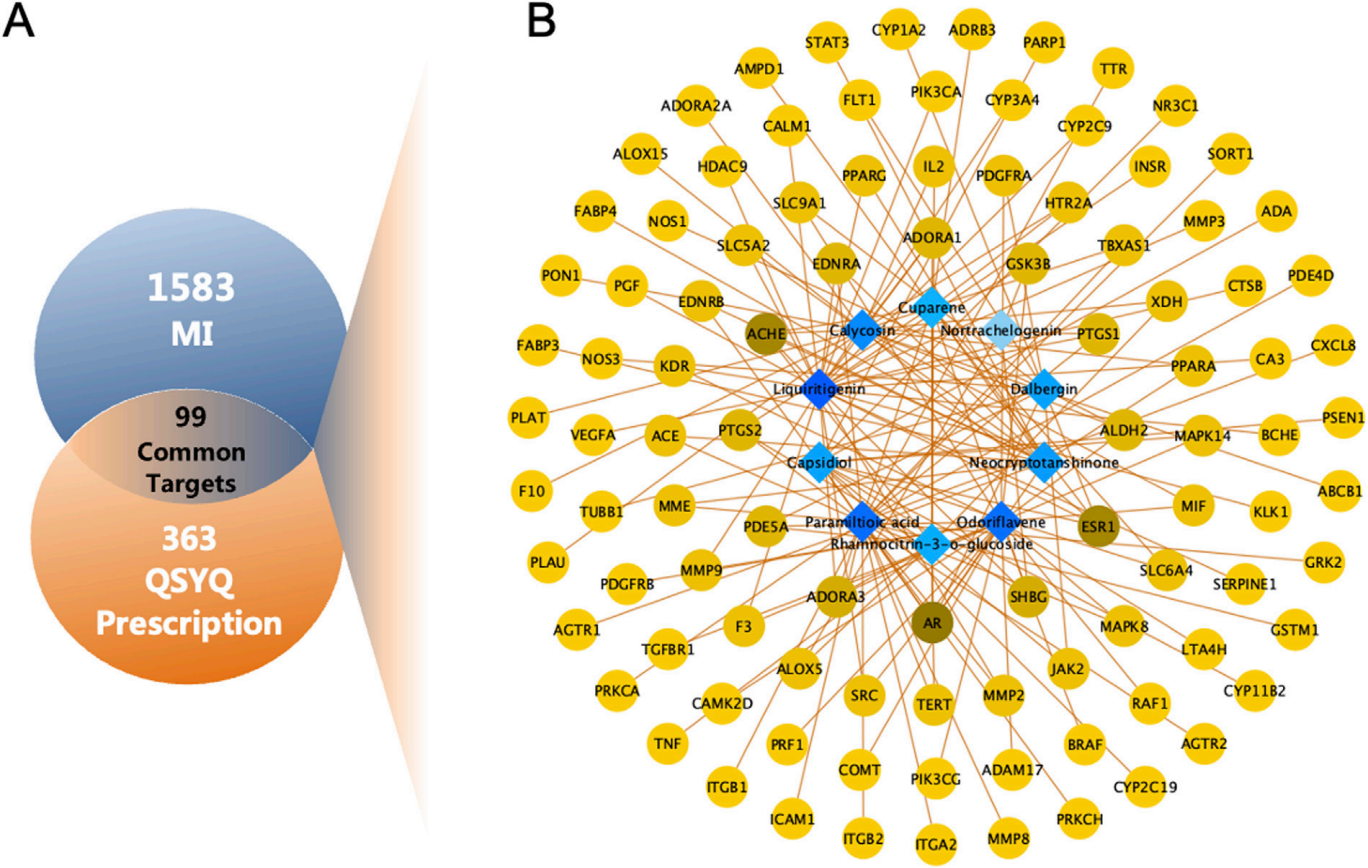
Network analysis of QSYQ treating MI. **(A)** Venn diagram of 99 common targets of QSYQ and MI. **(B)** C-T network of QSYQ. Blue nodes represent the active components; Yellow nodes indicate potential targets. Brown lines indicate the relations between the active components and the potential targets.

Excluding the three compounds (9,10-dimethoxy-pterocarpane-3-o-beta-d-glucoside, 9,10-dimethoxy-pterocarpan-3-o-β-d-glucopyranoside, paramiltioic acid) without therapeutic targets, 363 targets for 10 compounds were identified through TCMSP, SwissTargetPrediction, and DrugBank (Supplementary Table S2). After removing redundant entries, 1,583 potential targets for MI were obtained from Genecards, OMIM, and DisGeNET (Supplementary Table S3). To investigate potential targets of QSYQ in MI, the Venn diagram was drawn (Figure 3A). Finally, 99 consensus targets were identified to be possible therapeutic targets of QSYQ in the treatment of MI, which were employed to construct a Compound-Target network. Cytoscape was utilized to construct the Compound-Target network consisting of 109 nodes (10 components, 99 targets), and 156 Compound-Target interactions (Figure 3B; Supplementary Table S4).

For nodes within the constructed Compound-Target network, their topological feature degrees were calculated using network analysis. Nodes showing increased degrees compared with mean neighbor number (2.862) were detected and obtained as main hub networks. In total, 12 key targets of QSYQ in treating MI were identified due to their primary positions within the network (Table 2).

**TABLE 2:** Topological information of 12 key targets.

Swiss prot	Name	Description	Degree	Betweenness centrality	Average shortest path length	Closeness centrality
P10275	AR	Androgen receptor	6	0.171673586	2.462962963	0.406015038
P03372	ESR1	Estrogen receptor	5	0.089836282	2.666666667	0.375
P22303	ACHE	Acetylcholinesterase	5	0.05256812	2.740740741	0.364864865
P0DMS8	ADORA3	Adenosine receptor A3	4	0.031645643	2.814814815	0.355263158
P04278	SHBG	Sex hormone-binding globulin	4	0.031516462	3.055555556	0.327272727
P05091	ALDH2	Aldehyde dehydrogenase	3	0.028014476	3.018518519	0.331288344
P23219	PTGS1	Prostaglandin G/H synthase 1	3	0.050091814	2.685185185	0.372413793
P30542	ADORA1	Adenosine receptor A1	3	0.018871537	2.981481481	0.335403727
P35354	PTGS2	Prostaglandin G/H synthase 2	3	0.016439053	3.166666667	0.315789474
O76074	PDE5A	cGMP-specific 3′,5′-cyclic phosphodiesterase	3	0.01799077	3.148148148	0.317647059
P49841	GSK3B	Glycogen synthase kinase-3 beta	3	0.019337885	2.981481481	0.335403727
P25101	EDNRA	Endothelin-1 receptor	3	0.034891911	2.814814815	0.355263158

### 3.4 GO and KEGG enrichment analysis

**FIGURE 4:**
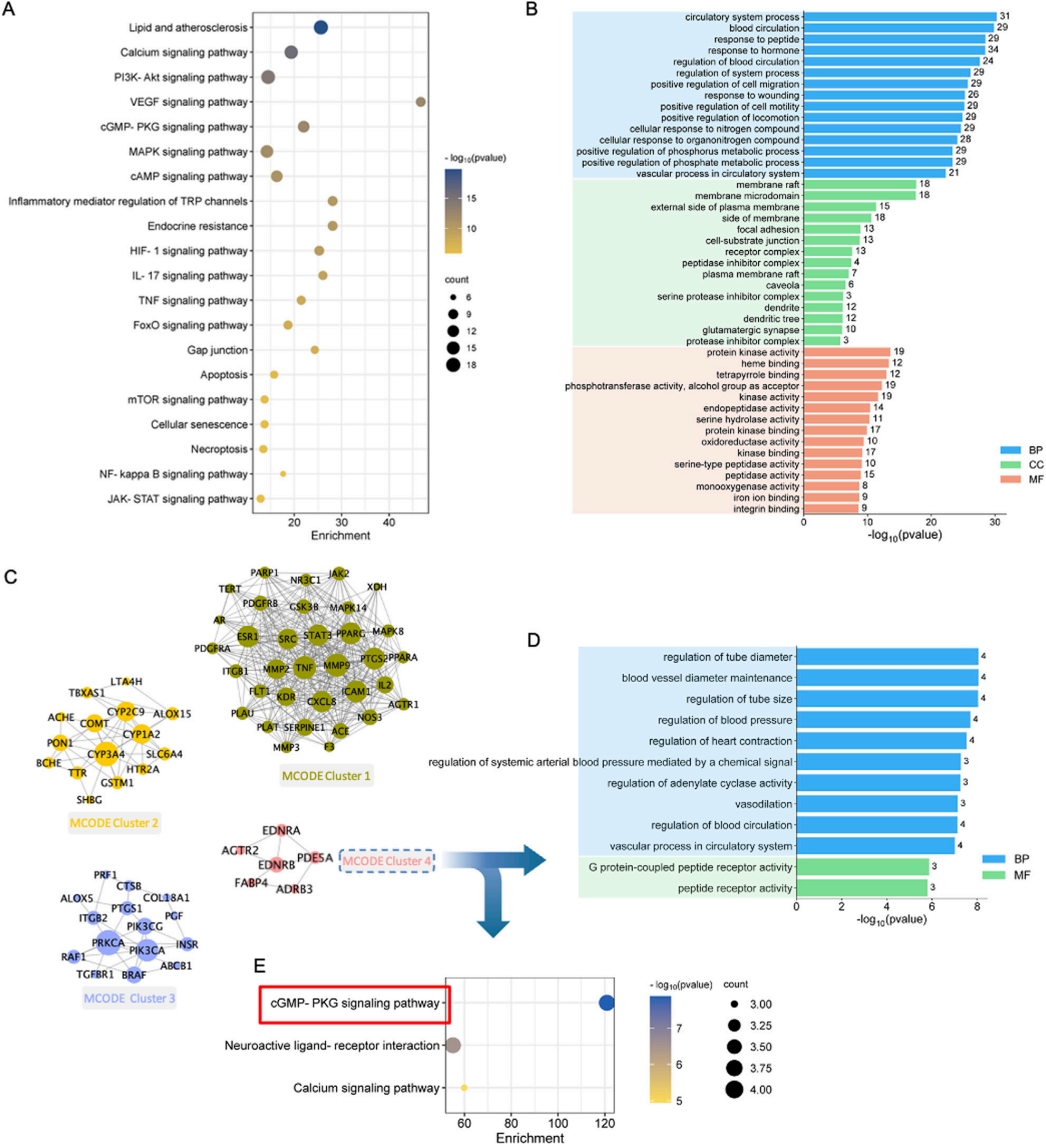
Enrichment and modular analysis of the key targets. **(A)** The top 20 significantly enriched terms in KEGG pathways associated with QSYQ against MI. **(B)** The top 15 significantly enriched terms in biological processes, molecular functions, and cellular components. **(C)** Derived different MCODE clusters: Green nodes were kernel genes in MCODE cluster1; Orange nodes were kernel genes in MCODE cluster2; Purple nodes were kernel genes in MCODE cluster3; Pink nodes were kernel genes in MCODE cluster4. **(D)** The top enriched terms of MCODE cluster4 in biological processes (blue parts) and molecular functions (light brown parts). **(E)** Significantly enriched terms of MCODE cluster4 in KEGG pathways.

To clarify the possible functions of the 99 targets, Metascape platform was used for gene annotation and functional enrichment (Figure 4; Supplementary Table S5). The 15 most significant GO-biological processes were associated with 3 major aspects of MI treatment, including regulation of circulatory system, protection of blood vessels, and adjustment of nitrogen compound. In addition, the 15 most significant GO-molecular functions included oxidoreductase activity, protein kinase activity, serine hydrolase activity, monooxygenase activity, and iron ion binding. The 15 most significant GO-cellular components included membrane raft, membrane microdomain, receptor complex, and protease inhibitor complex (Figure 4B). Those KEGG pathways enriched included energy metabolism-related pathways (including PI3K-Akt, cGMP-PKG, cAMP, and mTOR signaling pathways), immune and inflammation regulation-related pathways (like TNF, MAPK, NF-kappa B, and JAK-STAT pathways), endocrine resistance-related signaling pathways, and cell death-related pathways (like apoptosis, cellular senescence, and necroptosis) (Figure 4A).

### 3.5 MCODE enrichment

To deeply understand the possible mechanism, the molecular network was constructed using MCODE algorithm, which generated four significant clusters (Figure 4C; Supplementary Table S6). To investigate the possible target functions in distinct clusters, the targets of each cluster were uploaded into Metascape for functional enrichment, with the parameters set to “H species,” and the results were arranged based on the p-value. Therefore, Clusters 1, 2, and 3 were related to several biological process, cellular component, molecular function terms, and KEGG pathways (Supplementary Table S7). Enrichment of Cluster 4 was related to vasodilation and energy metabolism (Supplementary Table S8). Biological processes were associated with tube diameter regulation, heart contraction regulation, vasodilation, etc*.* The molecular functions included peptide receptor activity and G protein-coupled peptide receptor activity (Figure 4D). In addition, cGMP-PKG pathway was most significantly enriched (Figure 4E). Therefore, the important mechanism by which QSYQ treated MI might be related to promoting vasodilation and regulating energy metabolism.

### 3.6 QSYQ improved heart function in AMI rats

**FIGURE 5:**
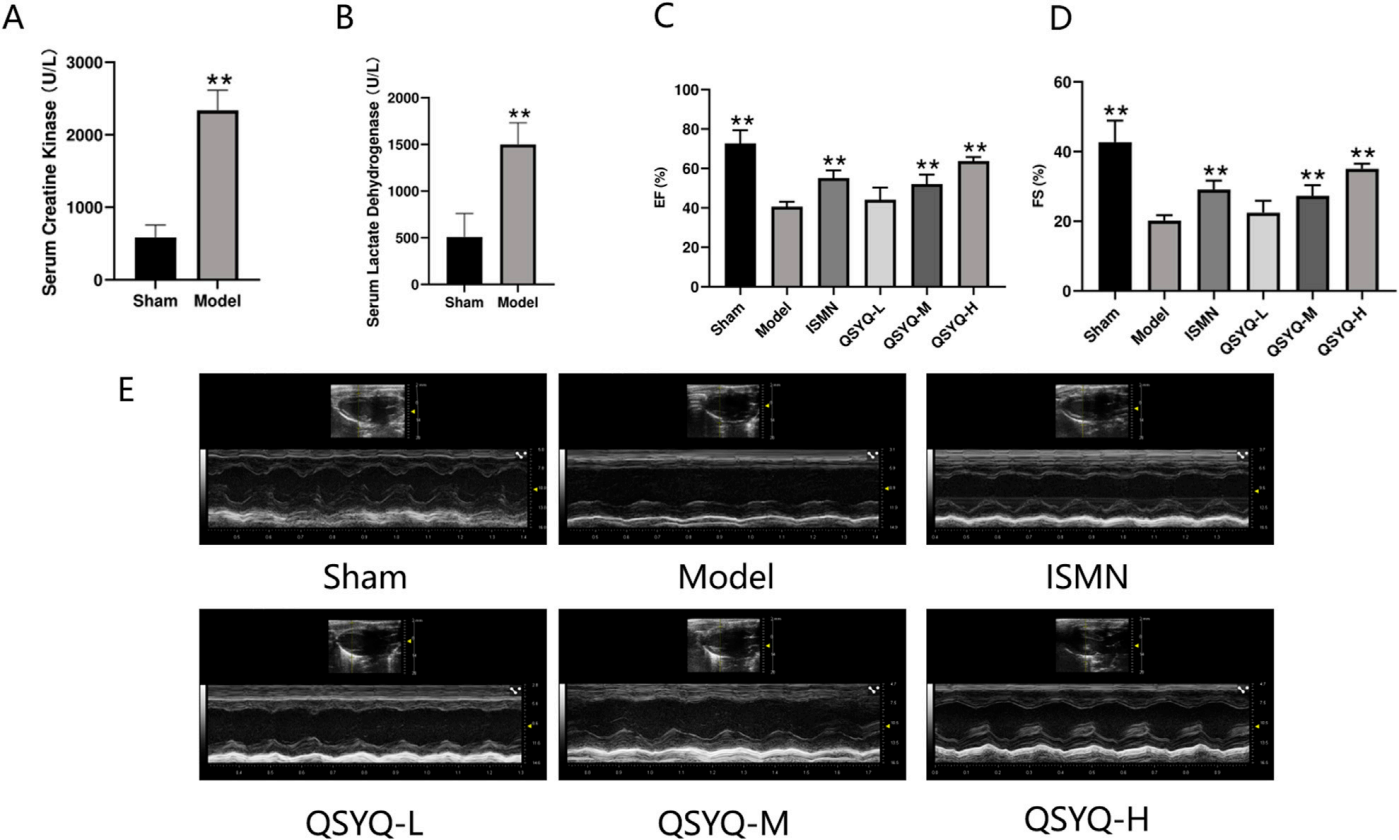
Construction of rat model and assessment of the therapeutic effects of QSYQ. **(A, B)** Concentration analysis of serum creatine kinase and serum lactic dehydrogenase. **(C)** Echocardiography-based assessment of EF. **(D)** Echocardiography-based assessment of FS. **(E)** 2D echocardiogram in sham, model, ISMN and QSYQ groups. Data are shown to be mean ± SD. **P < 0.01, in relative to the model group, evaluated using one-way ANOVA with Dunnett’s *post hoc* test.

MI promotes the acute increases in CK and LDH levels in circulation (Yu et al., 2021). To evaluate the efficacy of the MI rat model preparation, CK and LDH contents in serum were detected at 4 h postoperatively (Figures 5A, B). CK and LDH contents of MI group significantly increased compared with Sham group, suggesting the successful establishment of MI rat model. At 28 days after surgery, echocardiography revealed that EF and FS levels of model group significantly declined relative to sham group, demonstrating that heart function was severely impaired. After treated by QSYQ for 28 days, values of EF and FS were up-regulated in the middle(M)- dose, high(H)-dose and ISMN groups, indicating that QSYQ can improve cardiac function and reverse ventricular remodeling (Figures 5C–E).

### 3.7 QSYQ attenuated oxidative stress in MI rats

**FIGURE 6:**
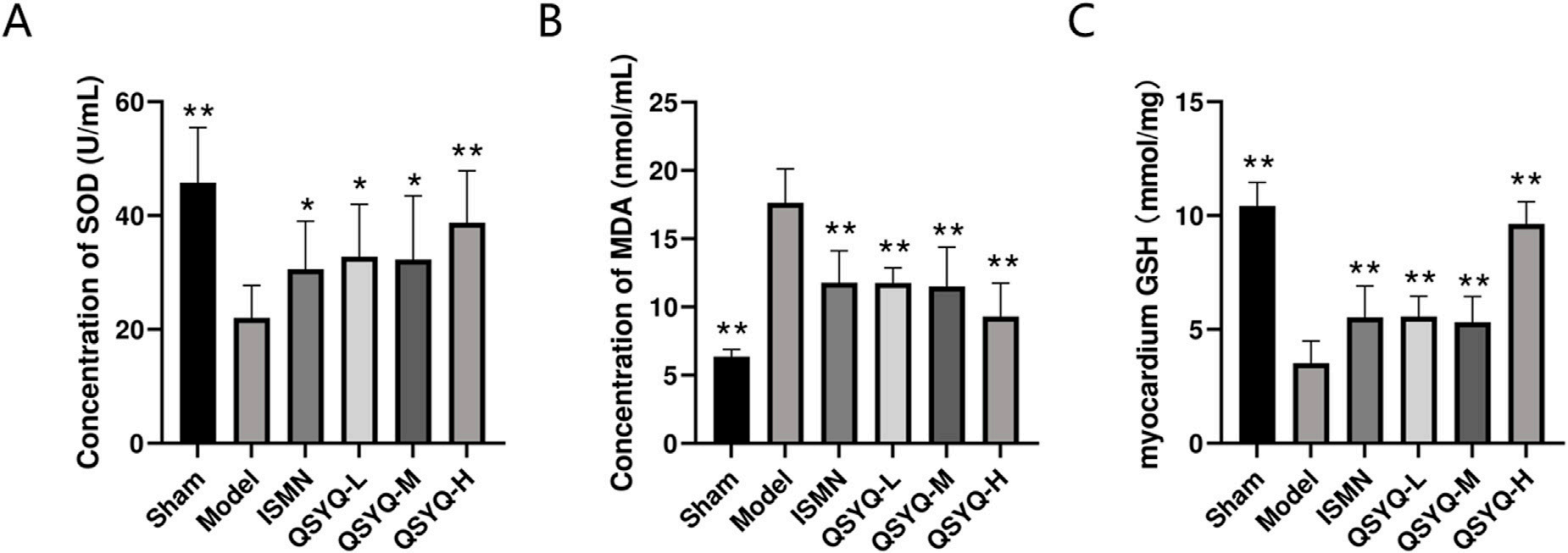
QSYQ can attenuate myocardial oxidative stress in MI rats. **(A)** Concentration analysis of myocardial tissue SOD. **(B)** Concentration analysis of myocardial tissue MDA. **(C)** Concentration analysis of myocardial tissue GSH. Data are represented to be mean ± SD. *P < 0.05, **P < 0.01, compared with the model group, evaluated using one-way ANOVA with Dunnett’s *post hoc* test.

The oxidative stress marker levels were evaluated in the rat myocardial tissue. Significantly increased SOD (Figure 6A) and GSH levels (Figure 6C), as well as significantly decreased levels of MDA (Figure 6B), were observed in rats of QSYQ (L-, M-, and H-dose) treatment and ISMN groups, compared with model group. QSYQ effectively mitigated oxidative stress in the heart tissues.

### 3.8 QSYQ mitigated NO-cGMP-PKG pathway and decreased eNOS uncoupling during MI

**FIGURE 7:**
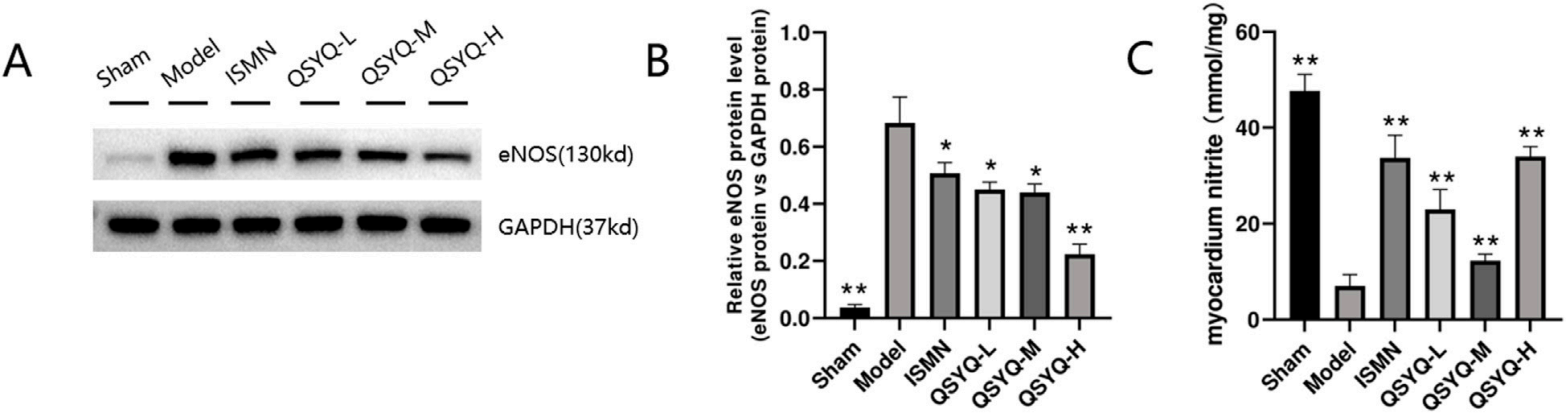
QSYQ can reduce eNOS uncoupling in MI rats. **(A, B)** Western blot analysis of eNOS monomer and phosphorylated monomeric isoformin myocardium. **(C)** Nitrite concentration in the myocardium. Data are shown to be mean ± SD. *P < 0.05, **P < 0.01, compared with the model group, evaluated through one-way ANOVA with Dunnett’s *post hoc* test.

**FIGURE 8:**
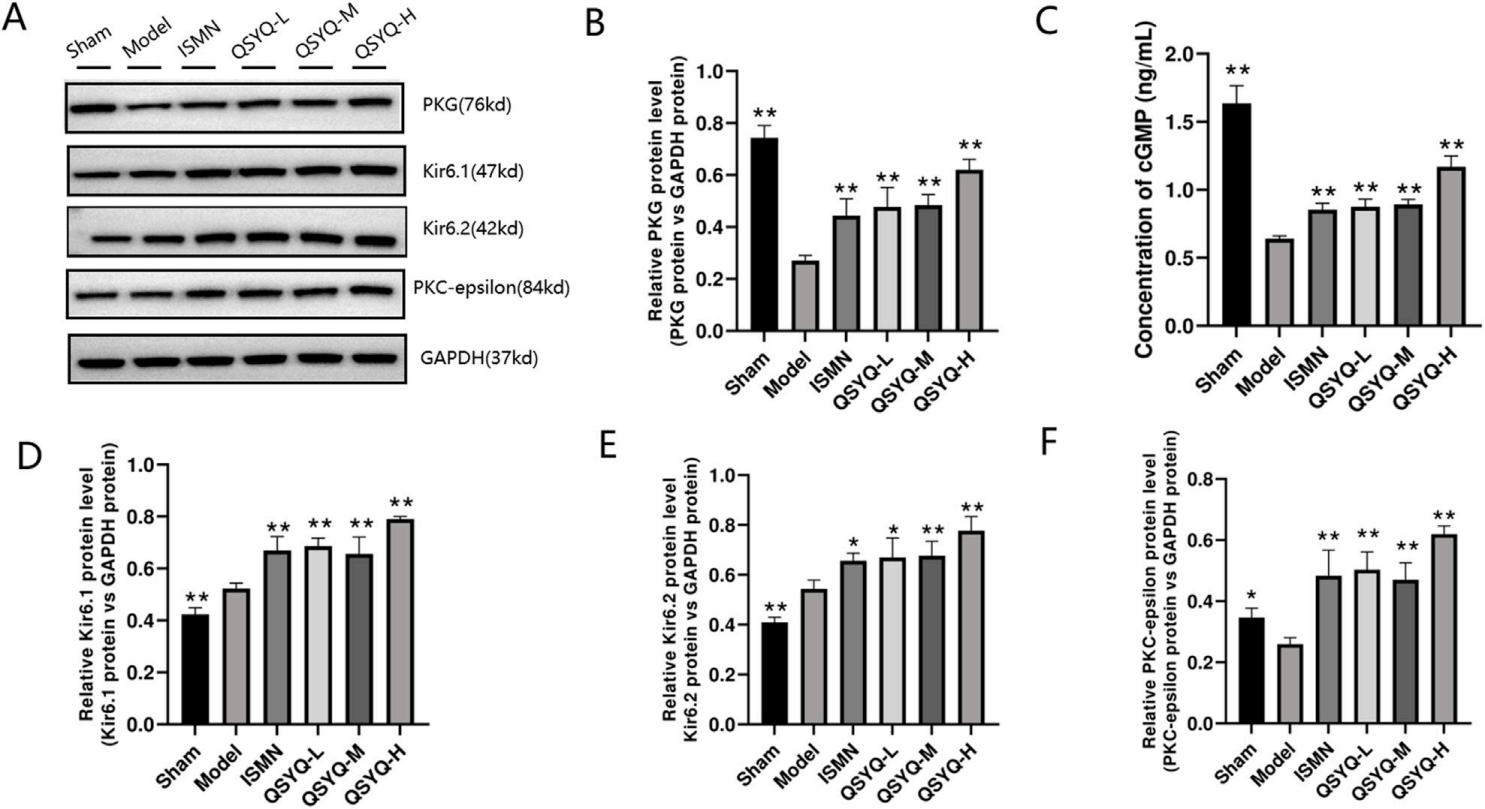
QSYQ can activate the NO-cGMP-PKG pathway and mitochondrial K_ATP_ in MI rats. **(A, B)** Western blot analysis of PKG. **(C)** cGMP content in plasma determined through ELISA. **(D–F)** Western blot analysis of two subunits (Kir6.1 and Kir6.2) of K_ATP_ and PKC-epsilon in myocardium. Data are shown to be mean ± SD. *P < 0.05, **P < 0.01, compared with the model group, evaluated through one-way ANOVA with Dunnett’s *post hoc* test.

The NO-producing eNOS dimer in the physiological environment will become a monomer after stimulating MI oxidative stress, thus generating a greater number of superoxide anions. According to our results, significantly decreased levels of eNOS (Figures 7A, B), were observed in rats of QSYQ (L-, M-, and H-dose) treatment and ISMN groups, compared with model group. NO-cGMP-PKG pathway plays a vital role in regulating cardiovascular function and promoting cardioprotection (Penna et al., 2017; Gáspár et al., 2020), particularly in cases of MI with diminished myocardial PKG activity. eNOS increases the arginase activity while decreasing NO generation in endothelial dysfunction (Shemyakin et al., 2012). According to our results, NO-cGMP-PKG pathway among MI rats was inhibited, as demonstrated by the reduced NO metabolites within myocardium (Figure 7C) and decreased PKG protein level (Figures 8A, B) QSYQ groups at different doses significantly recovered NO-cGMP-PKG pathway activation by promoting NO metabolites, PKG and cGMP in MI (Figures 8A–C).

QSYQ may induce cardioprotection by enhancing the opening and levels of K_ATP_ downstream of PKG. Afterwards, the QSYQ cardioprotection-related protein was detected. QSYQ activated mitochondrial K_ATP_ through PKG to trigger cardioprotection. Since K_ATP_ is a critical protective target for heart disease, we analyzed the protein levels of two K_ATP_ subunits (Kir6.1 and Kir6.2). Based on Western blot assay, Kir6.1 and Kir6.2 levels of model group significantly increased relative to the sham-operation group (Figures 8D, E). QSYQ (L-, M-, and H-dose) groups had obviously increased K_ATP_ subunit protein expression. Compared with model group, QSYQ (L-, M-, and H-dose) groups had significantly increased PKC-epsilon protein expression (Figure 8F). Thus, enhancing K_ATP_ opening and expression is a vital mechanism by which QSYQ resists MI.

## 4 Discussion

MI is associated with higher hospitalization and mortality rates, as well as dismal prognostic outcome, and may subsequently progress into heart failure, which has posed great burdens on the patients’ families and the society (Bahit et al., 2018). While evidence-based therapies can decrease the mortality rate among MI cases, the rehospitalization rate and cardiac risk are still high among these patients (Ko et al., 2020). Although the practicality of this commonly performed surgery has significantly advanced due to the maturation of diagnostic and treatment technologies, and the continuous enhancements in PCI for suitable patients, its impact on symptom alleviation is not substantial for individuals with single-vessel disease who are currently undergoing optimal drug treatment and experiencing a lower burden of angina pectoris (Al-Lamee et al., 2018). In China, TCM treatment strategies have been widely adopted as first-line clinical therapies, particularly in areas with limited medical resources, and have yielded favorable therapeutic outcomes. QSYQ has demonstrated significant clinical benefits in the treatment of cardiovascular diseases. Here, we combined UHPLC, network analysis and experimental validation to reveal the therapeutic effects, efficacy, and potential mechanisms of QSYQ against MI.

UHPLC has been widely employed for the analysis of TCM formulas. Considering the complexity of chemical ingredients contained in QSYQ, we opted to use UHPLC-Q-Orbitrap HRMS to analyze its chemical composition. This analytical technique disclosed 111 different compounds in QSYQ, The representative compounds identified include astragalus membranaceus isoflavone (e.g., calycosin, formononetin, calycosin 7-o-glucoside); astragalus membranaceus saponins (e.g., astragaloside I, II, III, IV, VI); danshen hydrosoluble composition (e.g., salvianolic acid A, B, G, J); danshen liposoluble constituent (e.g., neocryptotanshinone, 1,2,5,6-tetrahydrotanshinone); panaxadiol saponin (e.g., ginsenoside Rb1, Rb3, Rd, Rg3); panaxtrol saponin (e.g., notoginsenogside R1, R2, ginsenoside Rg1, Re); and oils and lipids (e.g., ethylpalmitate, ethyl pentadecanoate), many of which had been identified in previous studies (Han et al., 2015; Zhuo et al., 2017; Xie et al., 2019). Following ingestion, these compounds enter the gastrointestinal tract and are absorbed into the bloodstream, where certain constituents may require pharmacological activation to exert therapeutic effects on MI (Tang et al., 2022). Consequently, the thirteen components detected in rat serum were identified as potential active constituents of QSYQ with therapeutic efficacy against MI. Network analysis was employed to elucidate the complex mechanisms by which TCM exerts its therapeutic effects (Li and Zhang, 2013). Based on UHPLC analysis results, primary targets of QSYQ’s potential active components were cross-referenced with disease-related targets to identify potential therapeutic mechanisms for MI treatment. The potential active components of QSYQ may treat MI by acting on vasodilation, energy metabolism, and the cGMP-PKG signaling pathway. Previous research has demonstrated that the cardioprotective effects mediated by the cGMP-PKG signaling pathway are closely linked to vasodilation and energy metabolism. This pathway may serve as a potential therapeutic target for the treatment of MI (Inserte and Garcia-Dorado, 2015a). Follow-up experiments were conducted to validate these mechanisms.

In this study, QSYQ obviously mitigated the decline of heart function. The protection is probably achieved by activating NO-cGMP-PKG pathway and mitochondrial K_ATP_. The QSYQ-related mechanisms in MI management are probably associated with the reduction of oxidative stress while promoting vasodilation. Following MI, the elevation of mitochondrial reactive oxygen species (ROS) contributes to the increased fibrosis in the heart, contributing to a substantial rise in absolute scar volume and the serious cardiac systolic dysfunction (Janbandhu et al., 2022). It is demonstrated that mitigating oxidative stress can enhance coronary angiogenesis and improve cardiac function in patients with non-repercussed MI (Teixeira et al., 2023). Besides, the known cardiovascular risk factors are found to promote ROS production and reduce endothelial NO generation (Rochette et al., 2013). Critical molecular events during cardiovascular events including macrophage infiltration/activation, endothelial cell activation, and oxidative modification of phospholipids and lipoproteins, can be suppressed through endothelial NO and enhanced through vascular oxidative stress (Förstermann et al., 2017). Therefore, preventing vascular oxidative stress while improving endothelial NO generation is the promising treatment strategy in addition to managing the known risk factors.

NO is a principal effector molecule orchestrating vascular tone modulation and upholding vascular integrity, which can be predominantly synthesized and produced via vascular endothelial cells (Tejero et al., 2019). Its biochemistry can be complicated, while continuous emerging knowledge in controlling NO production and signaling mechanisms has exhibited its crucial impact on regulating cardiovascular activity, metabolism, immunity, and neurotransmission. Abnormal NO signaling has been identified as the vital characteristic of numerous main diseases, including diabetes, cancer and cardiovascular disease (Lundberg and Weitzberg, 2022). In mammals, the synthesis of NO involves three distinct isoforms of the eNOS, including neuronal “n”NOS, inducible “i”NOS, and endothelial “e”NOS (also called NOS I-III, separately). Among them, eNOS is the most crucial for maintaining blood vascular dilation, controlling blood pressure, and exerting various activities of vasoprotection and anti-atherosclerosis. While eNOS is not identified as the “disease gene,” numerous cardiovascular risk factors may induce eNOS uncoupling, oxidative stress, and vascular endothelial dysfunction (Förstermann and Sessa, 2012). By extravascular diffusion in the vascular smooth muscle cells, NO can combine with the receptor and activate the soluble guanylate cyclase (sGC)-cGMP-PKG pathway, promoting vascular endothelium homeostasis and cardiac remodeling (Cai et al., 2023). Moreover, NO can additionally permeate into cardiomyocytes located proximal to endocardial endothelial cells and coronary capillaries. NO-cGMP-PKG pathway activation within myocardium exerts activities including anti-hypertrophy, anti-fibrosis, and angiogenesis, thus impeding cardiac remodeling (Bryan, 2018; Farah et al., 2018). In a state of normal physiological equilibrium, organisms synthesize a requisite quantity of NO to uphold homeostasis. Nevertheless, under specific pathological conditions, vascular endothelial cells incur damage, which leads to diminished NO generation and perturbation of homeostatic equilibrium, thus exacerbating cardiovascular progression (Lundberg et al., 2015; Hill et al., 2021). Based on our results, which encompassed serum ELISA and cardiac tissue Western blot analyses to evaluate oxidative stress and NO levels in MI rats, QSYQ treatment successfully alleviated eNOS uncoupling within the myocardium of MI rats, contributing to an obvious reduction in oxidative stress. These outcomes conform to previous research findings (Wang et al., 2013; Han et al., 2019).

Meanwhile, NO can activate the major receptor, NO-sGC, in vascular smooth muscle cells, which generates the second messenger cGMP. A vital effect of cGMP on cardiovascular system refers to its mediated activation of cGMP-dependent protein kinase I, resulting in smooth cell relaxation through different pathways (Kraehling and Sessa, 2017). cGMP can combine with the intracellular receptors, translate NO signaling and stimulate PKG to induce changes of phosphorylation level, thereby exerting its specific effect. cGMP expression increases in cells, which can target cGMP-PKG to exert its physiological impacts. PKG is a primary kinase related to the transduction of physiological effects in the cardiovascular system of mammals (Park et al., 2018). Several investigations have indicated the existence of numerous downstream effectors in the cardiovascular system mediated by cGMP-PKG. An elevated cytosolic Ca^2+^ concentration is associated with heightened cardiomyocyte inotropy; nevertheless, it is concurrently recognized as a causative factor for lethal reperfusion injury in cardiomyocytes (Sellak et al., 2013; Inserte and Garcia-Dorado, 2015b). Moreover, it has been shown that the activation of PKG-I through cGMP mediates the opening of mitochondrial K_ATP_ channels located on the inner mitochondrial membrane. The ensuing heightened potassium influx leads to matrix alkalinization, contributing to an increased production of H_2_O_2_ in complex I. Increased H_2_O_2_ levels activate PKC-epsilon, then affording protection to cardiomyocytes against cell apoptosis by suppressing mitochondrial permeability transition pores (MPTP) opening (Sarre et al., 2005; Costa and Garlid, 2008; 2009). PKG, or cGMP-dependent protein kinase, is the serine/threonine protein kinase with broad expression across eukaryotic cells. It is recognized as the paramount downstream target of cGMP (Gorbe et al., 2010). By regulating diverse molecular signaling pathways, PKG instigates vasodilation within vascular smooth muscle cells (Casin and Calvert, 2021). In addition, cGMP-PKG pathway facilitates post-conditional protection, partially through postponing the normalization of low intracellular pH in the process of reperfusion, which may be achieved through PKG-dependent suppression on Na (+)/H(+)-exchanger (Inserte et al., 2011). Under physiological conditions, mitochondrial K_ATP_ was inhibited by intracellular adenosine triphosphate (ATP) and was in a closed state (Nichols and Nichols, 2006). However, during myocardial ischemia and hypoxia, substantial ATP decomposition into ADP occurs or ATP synthesis is reduced, which stimulates the opening of mitochondrial K_ATP_ channels (Maslov et al., 2022). This finding explains the observed upward trend of K_ATP_ in the model group within our study, which became more pronounced following QSYQ treatment.

Our ultimate results demonstrate that QSYQ robustly activates NO-cGMP-PKG pathway and the downstream mitochondria K_ATP_ channels comprehensively. Its primary target pathway is associated with inhibiting oxidative stress and promoting vasodilation. However, its potential mechanism of action may be intricately linked to mitochondrial energy metabolism, which warrants further exploration. More investigations are needed to analyze how QSYQ influences mitochondrial function of myocardial cells within the cardiovascular ailments.

## 5 Limitations

However, this study has several limitations. Firstly, serum pharmacochemistry was utilized to identify potential active components of QSYQ, with serum from normal rats serving as the carrier. Differences in drug absorption, distribution, metabolism, and excretion exist between normal and pathological states. Therefore, further research is required to compare the pharmacokinetics and pharmacokinetic/pharmacodynamic correlations of QSYQ in normal and MI-affected animals *in vivo*, along with relevant *in vitro* studies. These findings will help to elucidate the pharmacodynamic basis of QSYQ. The results presented here offer a preliminary foundation for future pharmacokinetic studies. Secondly, while *in silico* network analysis provided valuable preliminary insights into potential pharmacological targets of QSYQ, this approach has inherent limitations. The analysis involved complex mixtures, leading to many-to-many correlations that may lack specific pharmacological relevance. The predictive nature of computational methods, while useful for hypothesis generation, cannot substitute for empirical validation. To address these challenges, future studies should incorporate rigorous experimental validation to differentiate meaningful pharmacological effects from speculative predictions, ensuring a more reliable assessment of QSYQ’s therapeutic potential. Finally, we acknowledge that treatment with QSYQ likely triggers a complex response, with the reduction of oxidative stress via the NO-cGMP-PKG signaling pathway being just one facet. The NO-cGMP-PKG pathway also has potential in preventing cardiovascular damage by mediating the activation of K_ATP_ channels. While certain proteins associated with the mitochondrial K_ATP_ channel have been identified, their exact role in myocardial mitochondrial protection remains unclear. Further in-depth studies are required to elucidate the mechanisms by which QSYQ influences mitochondrial K_ATP_ pathways in the context of MI.

## 6 Conclusion

To conclude, in this study, we integrated serum pharmacochemistry with network analysis and experimentally confirmed the potential mechanism of QSYQ against MI. Our findings indicate that QSYQ reduces oxidative stress, promotes vasodilation, and shows a cardioprotection through the activation of NO-cGMP-PKG pathway and the mitochondrial K_ATP_ channel. Our results shed valuable lights on clinical application of QSYQ in cardioprotection.

## Data Availability

The datasets used and/or analyzed during the current study are available from the corresponding author on reasonable request.

## Glossary

ANOVA: analysis of variance
ATP: adenosine triphosphate
cAMP: cyclic adenosine monophosphate
CK: creatine kinase
cGMP: cyclic guanosine monophosphate
EF: ejection fraction
eNOS: enzyme nitric oxide synthase
FS: fractional shortening
GO: Gene Ontology
GSH: glutathione
ISMN: Isosorbide mononitrate
IU: international units
JAK-STAT: janus kinase-signal transducer and activator of transcription
**K**_**ATP**_: ATP-sensitive potassium channel
KEGG: Kyoto Encyclopedia of Genes and Genomes
LDH: lactate dehydrogenase
MAPK: mitogen-activated protein kinase
MCODE: Molecular complex detection
MDA: malondialdehyde
MI: myocardial infarction
MPTP: mitochondrial permeability transition pores
mTOR: mechanistic target of rapamycin
*m/z*: mass-to-charge ratios
NF-kappa B: nuclear factor kappa-B
NO: nitric oxide
OD: optical density
PI3K-Akt: phosphatidylinositol 3 kinase-protein kinase B
PKC-epsilon: protein kinase C epsilon
PKG: protein kinase G
PVDF: polyvinylidene fluoride
QSYQ: QiShenYiQi dripping pills
Rt: retention time
SEM: standard error of the mean
sGC: soluble guanylate cyclase
SOD: superoxide dismutase
TCM: traditional Chinese medicine
TNF: tumor necrosis factor
UHPLC-Q-Orbitrap HRMS: ultra high performance liquid chromatography-q exactive-orbitrap high resolution mass spectrometry
