# Supplementary material for: UHPLC-Q-Orbitrap HRMS and network analysis to explore the mechanisms of QiShenYiQi dripping pill for treating myocardial infarction
Source: Front Pharmacol. 2024 Nov 1;15:1443560. doi: 10.3389/fphar.2024.1443560 (PMC11563805; doi:10.3389/fphar.2024.1443560)

Supplementary Material

# Supplementary Figure 1. The base peak ions chromatograms of QSYQ solution detected in positive (A) and negative (B) modes.

**
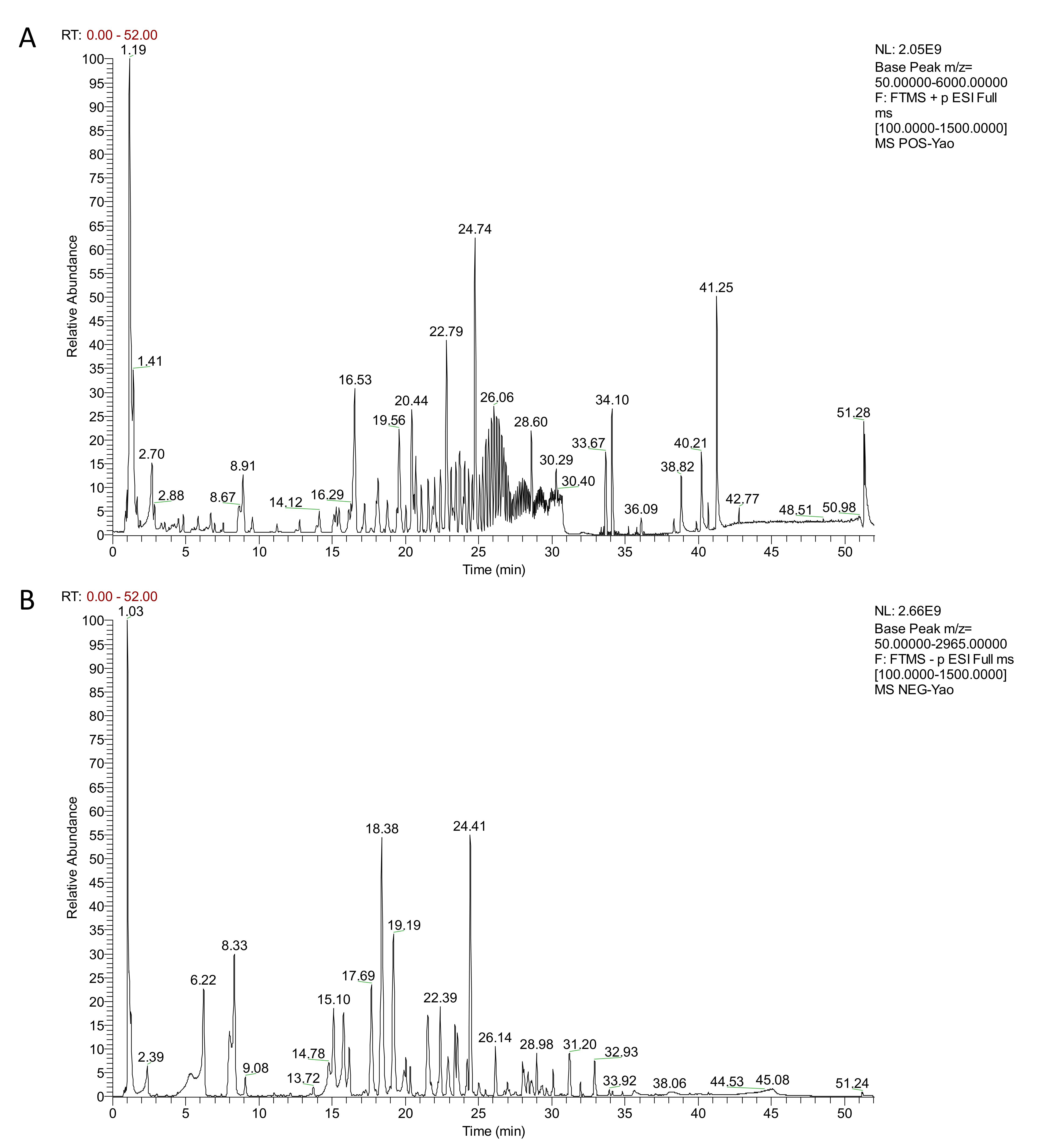
**

# Supplementary Figure 2. The base peak ions of blank serum detected in positive (A) and negative (B) modes.


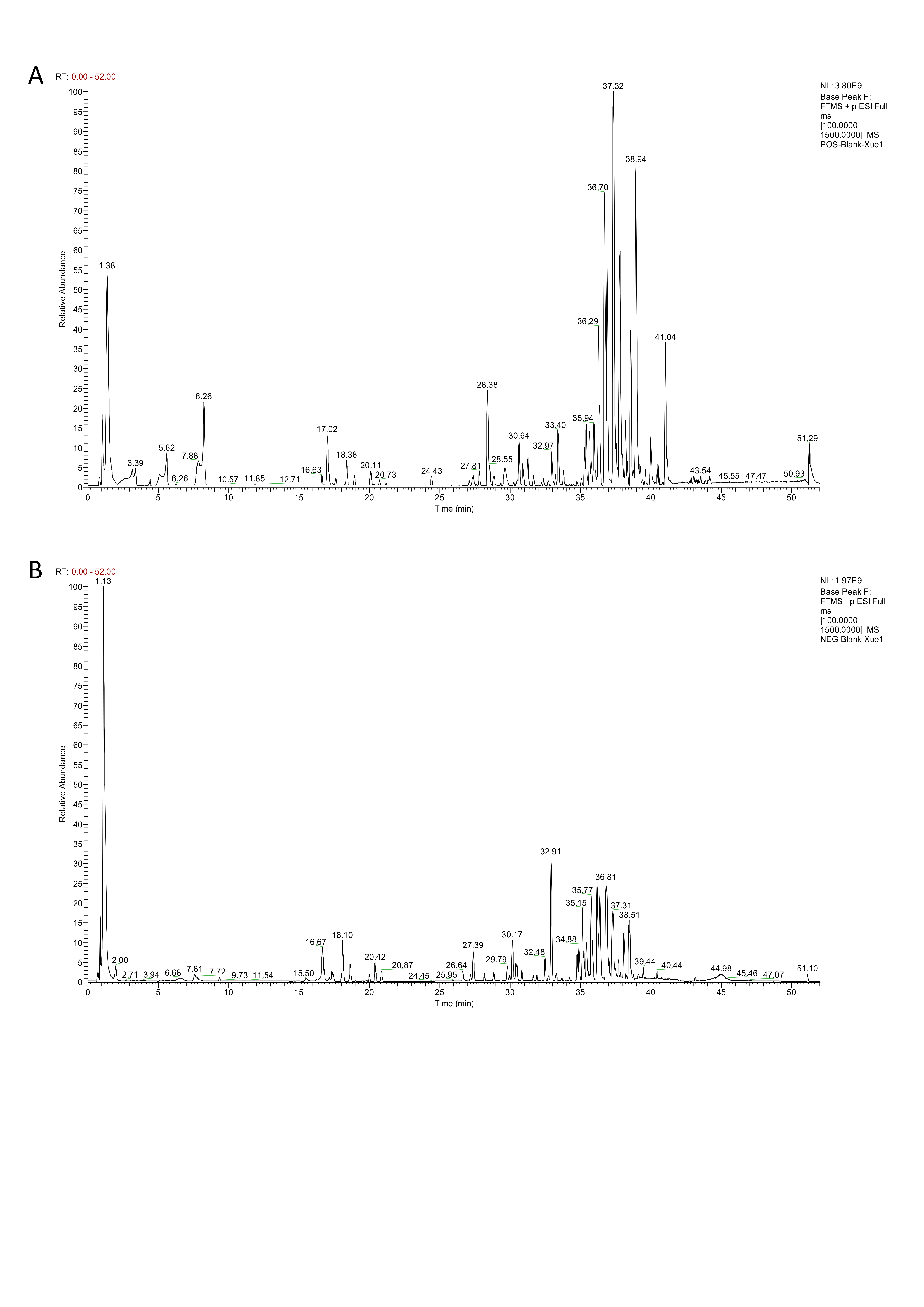

Supplement: Supplementary file 8 [file DataSheet1.docx]
